# Association Between Shift Patterns and Occupational Stress and Perceived Professional Benefits Among Nurses: A Cross‐Sectional Study

**DOI:** 10.1155/jonm/3178918

**Published:** 2026-06-18

**Authors:** Xianrong Kong, Longbiao Cai, Jia Wang

**Affiliations:** ^1^ TongJi Hospital, TongJi Medical College of HuaZhong University of Science and Technology, Department of Radiology, Wuhan, Hubei, China, tjh.com.cn

## Abstract

**Objective:**

This cross‐sectional study examined the associations between shift patterns, occupational stress, and perceived professional benefits among hospital nurses.

**Methods:**

A convenience sample of 325 nurses from a single tertiary hospital in Hubei Province, China, was enrolled. Data were collected using a general information questionnaire, a shift pattern scale, a perceived professional benefits scale, and an occupational stress scale. Statistical analyses included chi‐square tests, *t*‐tests, rank‐sum tests, Pearson’s correlation, and multivariable linear regression.

**Results:**

Nonshift nurses had significantly higher occupational stress scores than shift nurses (67.23 vs. 58.55, *p* < 0.001). Among shift nurses, those with 2–6 years of experience and those working > 6 night shifts per month reported higher stress levels. Among different shift patterns, only the 21:00–8:00 group scored lower in nurse–patient relationship than the nonshift group, whereas the 18:00–01:30 and 01:30–08:00 groups reported higher perceived professional benefits from positive occupational perceptions than the nonshift group. The nonshift group experienced higher occupational stress levels compared to the shift group. Pearson’s correlation analysis indicated that the overall score for occupational sense of gain was positively correlated with the total scores for work tasks and development, interpersonal support, patient care, and occupational stress. Positive but modest correlations were observed between several stress dimensions and professional benefit dimensions (*r* = 0.12–0.18, *p* < 0.05).

**Conclusions:**

In this single‐site sample, certain shift patterns and a higher monthly frequency of night shifts were associated with increased occupational stress, whereas longer shift experience was correlated with better nurse–patient relationship scores. Nursing managers may use these findings to limit night shifts to ≤ 6 per month, provide targeted support for mid‐career nurses, and monitor stress levels across shift types.

## 1. Introduction

Due to the specific demands of medical professions, most clinical nurses in hospital settings follow a shift‐based work schedule. Previous research has demonstrated that working under such a system is associated with various potential risks for nurses, including an increased vulnerability to cardiovascular diseases and lung cancer [1], as well as higher levels of occupational stress [2]. Occupational stress is generally defined as a functional impairment resulting from the interaction between individual characteristics and workplace conditions, whereby work demands exceed an individual’s coping capacity [3]. In contrast, perceived professional benefit is an emotional experience characterized by positive, pleasant, and satisfied feelings that nurses derive from their profession upon recognizing the benefits and advantages provided by their occupation [4]. This positive experience serves as an intrinsic motivational factor that can strengthen professional identity and mitigate job burnout, thereby promoting nurses’ mental health. Using a cross‐sectional, single‐site design, this study aimed to examine the current status of occupational stress and perceived professional benefits among nurses working under different shift schedules (nonshift group: long‐term fixed day shifts; shift group: various night shift patterns) and explore the associations between these factors. The findings are intended to provide nursing administrators with evidence‐based insights for designing healthier and more efficient shift schedules.

## 2. Subjects and Methods

### 2.1. Research Participants

Nurses currently employed at a Class A Grade 3 hospital in Hubei Province were recruited as research participants. Inclusion criteria for participants included the following: ① registered nurses with a minimum of one year of work experience at the hospital and ② provision of informed consent and voluntary participation in the study. The exclusion criteria included the following: ① nurses who were absent from the hospital due to sick leave, maternity leave, vacation, or other reasons; ② nurses who were trainees, interns, or on probation; and ③ nurses who were pregnant or lactating. Participants were recruited through the head nurses of various departments, following the principle of voluntariness.

### 2.2. Methods

#### 2.2.1. General Information Questionnaire

The questionnaire collected data on gender, age, years of professional experience, and departmental affiliation (Internal Medicine, Surgery, Obstetrics and Gynecology, Pediatrics, Emergency, Intensive Care Unit [ICU], Operating Room, Medical Technology Departments, and others). Professional designation was categorized as Nurse, Nurse Practitioner, Senior Nurse Practitioner, Deputy Chief Nurse Practitioner, or Chief Nurse Practitioner. Job classification was delineated as Clinical Role, Management Role, Research Role, or Teaching Role. Highest educational attainment was specified as secondary vocational education, higher vocational education, bachelor’s degree, or master’s degree and above. Employment status was classified as official establishment position, personnel agency employment, or contract‐based employment. Marital status was identified as married or unmarried, and number of children was categorized as none, one, or two or more. Monthly income was segmented into three brackets: < 5000 RMB, 5000–10,000 RMB, and > 10,000 RMB.

#### 2.2.2. Questionnaire on Nursing Shift Work

The shift work questionnaire addresses multiple dimensions, including whether the respondent participates in shift work, the duration of shift work tenure since joining the organization, the average interval between night shifts over the past year, the monthly frequency of night shifts, and the specific shift patterns employed. The identified shift patterns are as follows: shift pattern A (21:00–8:00), shift pattern B (18:00–01:30), shift pattern C (01:30–08:00), shift pattern D (17:00–02:00 + 06:00–08:00), shift pattern E (17:00–22:00 + 02:00–08:00), and standby shift.

#### 2.2.3. Nursing Job Stressors Scale

The nursing stressors scale, developed by Li and Liu [5], encompasses four primary dimensions that are closely related to occupational stress among nursing staff: work tasks and development (7–35 points), interpersonal support in the workplace (4–20 points), patient care (3–15 points), and nursing responsibility (5–25 points). The total score ranges from 19 to 95, with higher scores indicating greater occupational stress. The scale demonstrates high internal consistency, with a Cronbach’s *α* coefficient of 0.98.

#### 2.2.4. Nursing Professional Satisfaction Scale

The scale, revised by Hu and Liu [6], comprises five dimensions: positive occupational perception (3–15 points), good nurse–patient relationship (4–20 points), recognition from relatives and friends (3–15 points), sense of belonging to the team (3–15 points), and personal growth (4–20 points). The total score ranges from 17 to 85, with higher scores indicating greater contribution of these factors to promoting positive emotions among nursing staff. The Cronbach’s *α* coefficient for this scale is 0.95.

#### 2.2.5. Statistical Analysis

Data were double‐entered and cross‐checked, and a database was established using Excel. Statistical analyses were performed using SPSS Version 21.0 (IBM Corp., Armonk, NY, USA). Group comparisons were conducted using the chi‐square test, independent‐samples *t*‐test, or rank‐sum test as appropriate. Pearson’s correlation was used to examine bivariate associations between occupational stress and perceived professional benefits.

## 3. Results

### 3.1. Basic Information of the Research Subjects

A total of 350 questionnaires were distributed, and 325 valid responses were collected, yielding an effective response rate of 92.9%. Among the respondents, 65 were male and 260 female, with ages ranging from 23 to 58 years (mean age: 32.03 ± 6.14 years). Of these, 181 nurses participated in night shift rotations, while 144 did not. Work experience ranged from 1 to 35 years (mean: 8.31 ± 5.91 years). Educational background included the following: secondary vocational education (*n* = 27), higher vocational education (*n* = 57), bachelor’s degree (*n* = 213), and master’s degree or above (*n* = 28). Marital status included the following: married (*n* = 225), unmarried (*n* = 94), and other (*n* = 6). Professional ranks included the following: nurse (*n* = 101), nurse practitioner (*n* = 148), senior nurse practitioner (*n* = 61), and deputy chief nurse practitioner (*n* = 15).

### 3.2. Perceived Professional Benefits Across Shift Patterns

The results in Table 1 indicated that the score for the nurse–patient relationship dimension in the shift work group was 16.43 ± 2.85 points, which was significantly higher than that of the nonshift work group (15.51 ± 3.33 points), with a statistically significant difference (*p* < 0.05). Additionally, the nurse–patient relationship score for shift workers with more than 6 years of shift work experience was significantly higher than that of workers with less than 2 years and those with 2–6 years of shift work experience. The nonshift work group’s score was higher than that of the group with less than 2 years of shift work but lower than that of the group with more than 2 years of shift work, with statistically significant differences observed (*p* < 0.05). Furthermore, professionals adhering to shift pattern B (18:00–01:30) reported significantly more positive perceptions relative to other groups, whereas those working night shifts (21:00–8:00) had significantly lower scores compared to both nonshift workers and other shift groups (*p* < 0.05). No statistically significant differences were identified in other dimensions or in the total occupational benefits between personnel in shift and nonshift groups (Table 1).

**TABLE 1:** Comparison of job satisfaction dimensions among different shift groups.

	Positive occupational perception	Nurse–patient relationship	Family identification	Team cohesion	Self‐growth	Professional fulfillment
Nonshift group	11.53 ± 2.59	15.51 ± 3.33	11.42 ± 2.60	11.53 ± 2.59	15.30 ± 3.46	65.28 ± 13.42
Shift group	11.22 ± 2.23	16.43 ± 2.85	11.29 ± 2.29	12.02 ± 2.14	15.68 ± 2.86	66.64 ± 10.30
*p*	0.082	0.038	0.142	0.369	0.752	0.827

*Shift years*						
< 2	11.00 ± 2.26	14.26 ± 3.22	10.96 ± 2.46	10.87 ± 2.51	14.74 ± 2.93	61.83 ± 12.62
2–6	11.37 ± 2.35	16.61 ± 2.88	11.32 ± 2.36	12.16 ± 2.20	16.05 ± 2.82	67.51 ± 10.44
> 6	11.14 ± 2.12	16.87 ± 2.44	11.35 ± 2.20	12.22 ± 1.87	15.58 ± 2.84	67.16 ± 9.12
*p*	0.285	0.001	0.432	0.065	0.207	0.213

*Number of night shifts*						
< 2	11.39 ± 2.00	16.50 ± 2.83	11.67 ± 1.78	11.89 ± 1.97	15.78 ± 2.32	67.22 ± 9.30
2–6	11.06 ± 2.45	16.38 ± 2.93	11.12 ± 2.46	12.12 ± 2.13	15.63 ± 3.05	66.31 ± 11.02
> 6	11.40 ± 2.03	16.42 ± 2.80	11.40 ± 2.22	11.95 ± 2.23	15.73 ± 2.80	66.90 ± 9.88
*p*	0.354	0.274	0.449	0.812	0.988	0.99

*Shift patterns*						
A	11.12 ± 2.18	14.76 ± 3.36	11.18 ± 2.10	11.06 ± 2.59	15.12 ± 3.18	63.24 ± 12.13
B	13.44 ± 1.74	16.89 ± 1.62	11.89 ± 2.15	13.22 ± 1.30	17.44 ± 2.19	72.89 ± 6.83
C	11.71 ± 1.88	16.83 ± 2.62	11.45 ± 2.30	12.48 ± 1.88	16.24 ± 2.61	68.71 ± 9.30
D	11.21 ± 2.56	16.58 ± 3.33	11.15 ± 2.60	12.09 ± 2.42	15.82 ± 2.92	66.85 ± 11.44
E	10.71 ± 2.19	16.29 ± 2.59	11.21 ± 2.25	11.90 ± 1.96	15.26 ± 2.95	65.37 ± 9.75
Standby	10.92 ± 2.11	17.33 ± 3.11	11.25 ± 2.34	11.42 ± 2.57	15.17 ± 2.55	66.08 ± 11.37
*p*	0.009	0.045	0.651	0.118	0.158	0.198

### 3.3. Occupational Stress Across Shift Patterns

Nonshift nurses had higher total occupational stress and higher scores on all four dimensions (work tasks and development, interpersonal support, patient care, and nursing responsibility) compared to shift nurses (all *p* < 0.001). Within the shift group, nurses with 2–6 years of experience reported higher stress levels across all dimensions than those with < 2 years or > 6 years of experience (all *p* < 0.01). Nurses working > 6 night shifts per month had higher total occupational stress and higher dimension scores than those with ≤ 6 night shifts per month (all *p* < 0.01). Among shift patterns, pattern B (18:00–1:30) was associated with the highest total stress and highest scores on all dimensions, while pattern A (21:00–8:00) showed the lowest scores on several dimensions compared to other shift patterns (*p* < 0.001 for overall pattern comparison). The relevant results are presented in Table 2.

**TABLE 2:** Comparison of scores across various dimensions of occupational stress.

	Work tasks and development	Work interpersonal support	Patient care	Nursing responsibility	Total occupational stress
Nonshift group	25.32 ± 6.65	14.25 ± 3.93	10.67 ± 2.84	16.99 ± 5.12	67.23 ± 17.30
Shift group	23.09 ± 6.09	13.07 ± 3.45	9.59 ± 2.57	12.80 ± 4.76	58.55 ± 14.44
*p*	< 0.001	0.001	< 0.001	< 0.001	< 0.001

*Shift years*					
< 2	22.26 ± 6.96	12.26 ± 3.73	9.48 ± 2.98	13.83 ± 5.04	57.83 ± 17.73
2–6	23.86 ± 6.07	13.24 ± 3.37	9.70 ± 2.27	12.86 ± 4.82	59.66 ± 13.96
> 6	22.56 ± 5.84	13.13 ± 3.46	9.52 ± 2.74	12.44 ± 4.62	57.65 ± 13.97
*p*	0.002	0.006	0.001	< 0.001	< 0.001

*Number of night shifts*					
< 2	21.89 ± 5.52	12.56 ± 4.15	9.61 ± 3.24	11.72 ± 3.75	55.78 ± 14.60
2–6	22.50 ± 6.71	12.87 ± 3.58	9.60 ± 2.67	11.95 ± 5.01	56.92 ± 15.43
> 6	24.00 ± 5.47	13.36 ± 3.16	9.55 ± 2.31	13.92 ± 4.51	60.83 ± 13.14
*p*	0.002	0.005	0.001	< 0.001	< 0.001

*Shift patterns*					
A	21.24 ± 6.91	11.41 ± 2.76	8.82 ± 2.56	15.06 ± 4.72	56.53 ± 15.85
B	28.00 ± 7.65	15.89 ± 3.86	12.22 ± 2.39	18.33 ± 5.34	74.44 ± 18.01
C	23.1 ± 5.33	12.88 ± 3.13	9.36 ± 2.41	13.26 ± 4.6	58.6 ± 13.61
D	21.94 ± 4.99	13.03 ± 3.30	9.91 ± 2.42	11.73 ± 4.24	56.61 ± 12.43
E	23.26 ± 6.11	13.25 ± 3.54	9.37 ± 2.39	11.57 ± 4.41	57.46 ± 13.53
Standby	24.17 ± 7.66	13.00 ± 4.20	9.92 ± 3.68	13.75 ± 4.52	60.83 ± 17.87
*p*	< 0.001	0.001	< 0.001	< 0.001	< 0.001

### 3.4. Correlation Between Occupational Stress and Perceived Professional Benefits

Pearson’s correlation analysis showed that the total perceived professional benefits score was positively correlated with the scores for work tasks and development (*r* = 0.142, *p* = 0.014), interpersonal support at work (*r* = 0.172, *p* = 0.003), patient care (*r* = 0.167, *p* = 0.004), and total occupational stress (*r* = 0.145, *p* = 0.012). In addition, positive occupational perception was correlated with work tasks and development (*r* = 0.148, *p* = 0.009), interpersonal support (*r* = 0.177, *p* = 0.002), patient care (*r* = 0.174, *p* = 0.003), and nursing responsibility (*r* = 0.147, *p* = 0.010). Nurse–patient relationship scores were correlated with work tasks and development (*r* = 0.114, *p* = 0.048), interpersonal support (*r* = 0.125, *p* = 0.030), and patient care (*r* = 0.120, *p* = 0.036). Detailed correlation coefficients are presented in Table 3.

**TABLE 3:** Pearson’s correlation coefficients between occupational stress and diverse dimensions of perceived occupational benefits.

	Positive occupational engagement	Nurse–patient interactions	Family identity	Team collaboration	Personal development	Career satisfaction score
Work tasks and development	0.148^∗∗^	0.114^∗^	0.092	0.135^∗^	0.137^∗^	0.142^∗^
Work interpersonal support	0.177^∗∗^	0.125^∗^	0.150^∗∗^	0.176^∗∗^	0.141^∗^	0.172^∗∗^
Patient care	0.174^∗∗^	0.120^∗^	0.157^∗∗^	0.137^∗^	0.151^∗∗^	0.167^∗∗^
Nursing responsibility	0.147^∗∗^	−0.003	0.107	0.016	0.043	0.066
Total occupational stress	0.176^∗∗^	0.093	0.131^∗^	0.121^∗^	0.125^∗^	0.145^∗∗^

^∗^ *p* < 0.05.

^∗∗^ *p* < 0.001.

## 4. Discussion

### 4.1. Nurse–Patient Relationship

The nurse–patient relationship is a key element in nursing practice, and extensive research has explored its impact on care quality and patient satisfaction. For instance, Molina‐Mula and Gallo‐Estrada [7] reported that a strong nurse–patient relationship can shorten hospital stays, enhance care quality, and elevate patient satisfaction. In the present study, shift workers scored higher on the nurse–patient relationship dimension than nonshift workers. Among shift workers, those with more than six years of shift experience achieved the highest nurse–patient relationship scores. This pattern is consistent with the findings of Xue and Liu [8]. One possible explanation, though not directly tested here, is that longer shift experience may allow nurses to accumulate positive interaction skills and become more adept at managing patient relationships. Nurses beginning shift work may initially struggle with work–life balance, which could temporarily affect their relational skills. A recent study by Chen et al. [9] also found that nurse–patient communication quality improved significantly after three years of shift experience, supporting the role of experience accumulation. Moreover, Kim et al. [10] demonstrated that nurses with longer shift tenure reported higher empathy scores, which directly enhanced the nurse–patient relationship. Given the cross‐sectional design, we cannot infer causality; other unmeasured factors (e.g., personality and ward culture) may also contribute.

### 4.2. Different Night Shift Patterns

Among the six shift patterns examined, only pattern A (21:00–8:00) was associated with a lower nurse–patient relationship score compared to the nonshift group. In contrast, the other shift patterns had scores equal to or higher than the nonshift group. Nonshift nurses in this sample were primarily involved in administrative tasks (e.g., processing medical orders and medication management), which involve less direct patient contact. During the 21:00–8:00 shift, most patients are asleep, limiting opportunities for nurse–patient interaction. Thus, shift patterns that allow more awake patient contact may be associated with higher relationship scores. This observation aligns with Schäfer et al. [11], who found that longer working hours and consultation time contribute to improved patient care. For the 21:00–8:00 shift, nursing managers might consider implementing structured evening care and presleep rounds to enhance meaningful patient interaction [12]. Wang et al. [13] further demonstrated that adding a 30‐minute evening care round significantly improved patient satisfaction scores for the night shift, which could be a practical strategy. In addition, a qualitative study by Ozdemir and Kaplan [14] revealed that nurses on later night shifts felt isolated from patients, which reduced their perceived professional benefits; this aligns with our finding for pattern A. Again, these associations should not be interpreted as causal.

### 4.3. Positive Occupational Perception

Nurses working shift pattern B (18:00–1:30) reported higher positive occupational perception scores than nurses in other shift patterns. Pattern C (1:30–8:00) was associated with intermediate scores. One possible explanation is that early night shifts may allow nurses to maintain a more regular sleep schedule and better adjust their mental state, whereas late night shifts may disrupt circadian rhythms more severely [15]. Shift work has been shown in previous studies to be associated with reduced work engagement and occupational self‐efficacy, as well as increased burnout, depression, and anxiety [16]. Such mental health challenges could, in turn, influence nurses’ positive perception of their profession. Liu et al. [17] reported that perceived professional benefits partially mediated the relationship between shift work and mental health outcomes, suggesting that enhancing positive perceptions could buffer some negative effects. Furthermore, a cross‐cultural study by Zhao et al. [18] found that nurses who perceived greater autonomy in choosing shift patterns reported higher positive occupational perception, highlighting the importance of scheduling flexibility. Therefore, a well‐structured shift schedule that minimizes circadian disruption may be correlated with more positive professional perceptions. However, our cross‐sectional data cannot establish whether shift patterns cause differences in occupational perception or whether nurses with more positive attitudes self‐select into certain shift patterns.

### 4.4. Occupational Stress

In this study, the nonshift group had higher occupational stress scores than the shift group. Among shift nurses, those with 2–6 years of experience reported higher stress than those with < 2 years or > 6 years. Additionally, nurses working > 6 night shifts per month had higher stress scores than those with fewer night shifts. Shift pattern B (18:00–1:30) was associated with the highest stress levels. These findings are consistent with previous reports. Alrawashdeh et al. [19] identified 1–3 night shifts per week and ≥ 3 night shifts per week as independent risk factors for occupational burnout. Lin et al. [20] found that nurses who rarely or never participated in shift scheduling and those who worked overtime at least three times per week experienced higher job stress. Şanlıtürk [21] reported that ICU nurses with 4–6 years of experience had significantly higher job stress than those with ≥ 13 years of service. A large multicenter study by Sun et al. [22] confirmed that night shift frequency > 6 per month was associated with 40% increased odds of high occupational stress after controlling for confounders, which strongly supports our bivariate findings. In addition, a systematic review by Dall’Ora et al. [23] concluded that quick returns (short rest periods between shifts) were a major contributor to stress, which may explain why pattern B (ending at 1:30) was particularly stressful, as it often precedes an early start. Our results mirror these patterns. Taken together, higher night shift frequency and intermediate shift experience appear to be associated with elevated occupational stress, while longer experience may be associated with lower stress, possibly due to improved coping strategies.

### 4.5. Correlations Between Stress and Professional Benefits

We observed positive correlations between several dimensions of occupational stress (work tasks and development, interpersonal support, and patient care) and dimensions of perceived professional benefits (positive occupational perception, nurse–patient relationship, team cohesion, and personal growth). The total occupational stress score was also positively correlated with the total professional benefits score. These correlational findings may seem counterintuitive, but similar patterns have been reported elsewhere [24–26]. One interpretation, offered cautiously, is that some types of work pressure may coexist with higher engagement or professional fulfillment, rather than representing a simple linear “stress is harmful” relationship. Alternatively, these positive correlations could reflect the influence of unmeasured third variables (e.g., overall work motivation and ward leadership). Zhang et al. [27] observed a similar paradoxical correlation between workload and professional satisfaction among emergency nurses, and they suggested that moderate work challenges may enhance feelings of competence when accompanied by adequate support. A recent longitudinal study by Hu et al. [28] found that occupational stress and professional benefits shared a bidirectional positive relationship over time, possibly because nurses who are more committed to their work simultaneously experience both higher pressure and higher rewards. Because our data are cross‐sectional, we cannot determine the direction or mechanism of these associations. We also note that the correlation coefficients are small to moderate (*r* = 0.12–0.18), indicating that shared variance is limited. Future longitudinal research is needed to disentangle these relationships.

### 4.6. Implications for Nursing Management

Based on the observed associations, nursing managers may consider the following practical strategies:

1. Limit monthly night shifts to ≤ 6 for rotating nurses because > 6 night shifts per month were associated with higher occupational stress.
2. Provide targeted support for nurses with 2–6 years of shift experience, as this subgroup reported the highest stress levels. Support could include stress management workshops, peer mentoring, or regular check‐ins.
3. Redesign the 21:00–8:00 shift by incorporating structured nurse–patient interaction activities (e.g., evening comfort rounds and bedtime assessments) to improve the nurse–patient relationship, which was lowest for this shift pattern.
4. Monitor nonshift nurses (e.g., those in administrative or day‐duty roles), as they showed higher occupational stress than shift workers in this sample. Offering regular stress assessments and opportunities for patient interaction may be beneficial.
5. Use shift pattern B (18:00–1:30) cautiously. Although it was associated with high professional benefits, it was also associated with the highest occupational stress. This pattern may be best suited for experienced nurses who have developed effective coping strategies, and it should be paired with adequate rest periods.

### 4.7. Limitations

Several limitations should be acknowledged. First, the cross‐sectional, single‐site design precludes causal inference. Second, the analyses are based primarily on group comparisons and bivariate correlations; potential confounding variables such as age, years of experience, department, professional rank, and educational background were not controlled for in the main analyses. Third, the sample was drawn from one tertiary hospital in Hubei Province, which may limit generalizability to other regions or hospital levels. Fourth, all data were self‐reported, raising the possibility of common method bias. Fifth, multiple comparisons were performed without formal adjustment, so some significant findings may be due to chance. Sixth, the response rate (92.9%) is acceptable, but nonrespondents may differ systematically from respondents. Future studies should employ prospective cohort designs with stratified multisite sampling to confirm and extend these findings.

## 5. Conclusions

In this cross‐sectional study of 325 nurses from a single tertiary hospital, different shift patterns were associated with varying levels of occupational stress and perceived professional benefits. Nonshift nurses and those working shift pattern B (18:00–1:30) showed higher occupational stress scores. Longer shift experience was associated with better nurse–patient relationship scores. While some positive correlations between stress and professional benefits were observed, these associations were modest and should not be interpreted as causal. Nursing managers can use these findings to optimize shift schedules, provide targeted support for mid‐career nurses, and monitor stress levels across shift types.

## Author Contributions

Xianrong Kong conceived and coordinated the project. Xianrong Kong and Longbiao Cai searched and sorted the literature. Xianrong Kong and Jia Wang wrote the manuscript. Jia Wang undertook the final correction and refinement work.

## Funding

This research received no specific grant from any funding agency in the public, commercial, or not‐for‐profit sectors.

## Disclosure

All authors approved the submission.

## Ethics Statement

This study was approved by the Ethics Committee of Tongji Hospital, Tongji Medical College, Huazhong University of Science and Technology. All procedures were conducted in accordance with the Declaration of Helsinki. Written informed consent was obtained from all participants prior to data collection.

## Conflicts of Interest

The authors declare no conflicts of interest.

## Data Availability

The datasets generated and analyzed during the current study are available from the corresponding author upon reasonable request.
